# Prof. Clay’s New Cancer Cure

**Published:** 1880-08

**Authors:** Edmund Andrews

**Affiliations:** Professor of Surgery, Chicago Medical College; No. 6 Sixteenth St., Chicago


### ARTICLE III.

PROF. CLAY'S NEW CANCER CURE. By EDMUND ANDREWS, A. M., M. D., Professor of Surgery, Chicago Medical College.

Widespread attention is being attracted on both sides of the Atlantic by a remarkable new series of experiments on the treatment of cancer by internal administration, recently published by Prof. John Clay, of the Queen's Hospital, Birmingham, England.

The alleged curative agent, a mixture of Chian turpentine and flowers of sulphur, bids fair, for a time at least, to rival the once famous Cundurango in its reputation as a cancer specific. Concerning its value in this disease, Prof. Clay makes the following assertions, supporting them by a number of cases: "The turpentine seems to act upon the periphery of the growth with great vigor, causing the speedy disappearance of what is usually termed the cancerous infiltration, and thereby arresting the further development of the tumor. It produces equally efficient results on the whole mass, seemingly destroying its vitality, but more slowly. It appears to dissolve all the cancer cells, leaving the vessels to become subsequently atrophied and the firmer structures to gradually gain a comparatively normal condition.

"It is a most efficient anodyne, causing an entire cessation of pain in a few days, and far more effectually than any sedative I have ever given. In the cases I have described no sedative was given in any instance, although in some cases, where great pain had existed previously to commencing the treatment, large doses had been given. Whether this arrest of pain arises from the death of the tumor or, as my son suggests, is due to there being no longer irritation of the sentient nerves (in consequence of the tension being withdrawn by the removal of the cells), the fact is the same.

"If, after the use of the remedy for some weeks, one of these cases were examined by a stranger for the first time he would probably conclude that it was one of commencing malignant disease, by reason of the irregularities of the surface. The effect of the remedy being first to remove the cellular structures, any loss of tissue produced by the invasion of the disease cannot be restored, and hence the irregular touch and appearance, even after cicatrization. The arrest of the hæmorrhagic discharge, and the remarkable freedom from glandular affections, after a lengthened use of the turpentine, are especially important factors in materially aiding the removal of the cachexia, and of improving the general condition of the patient.

"Without being in a position to affirm that the Chian turpentine is a positive cure for advanced cancer of the female generative organs, yet, however the facts here adduced may be interpreted in this respect, two circumstances are indisputable—one, that all the patients, after several months' treatment, are living, and that the disease has not advanced, as is usually the case, but has retrogressed—in fact has all but disappeared; and it may at least be safely asserted that when the remedy is steadily used for some time, it arrests the progress of the disease, and relieves the pain incidental to the morbid growth in a manner which cannot be said of any other remedy."

The daily papers have reproduced Prof. Clay's articles, and the laity as well as surgeons are making vigorous demands for the drug. Persons in Chicago, without waiting for medical advice, have telegraphed to New York and even London to procure it. Under these circumstances, the question of the genuineness of the agent becomes of great importance.

Telegraphic inquiry of a leading and reliable house in New York has assured me that there is no genuine Chian turpentine in this country. The article sold under that name by some of the Eastern firms is a fraud, and consists of Venice turpentine flavored with aromatics. The *Chemist and Druggist*, a London pharmaceutical journal, asserts that there is very little of the genuine in market in that city, and publishes letters from the vicinity where the substance has been procured heretofore, showing that now there is none to be obtained either in Athens or Smyrna, except some fraudulent imitations.

Meantime there was resurrected from the Apothecaries' Hall in London an old cask containing fifty pounds of the genuine article, which had lain untouched for nearly half a century. This was quickly sold out at fabulous prices, so that, as the *Chemist*

*and Druggist* remarks, the cask "proved a small gold mine." A gentleman in Chicago, who has influential acquaintances in London, has obtained a small amount of the genuine article from the same stock that supplied Prof. Clay. As its identity is perfectly proved, and it exactly corresponds to all the tests mentioned by Prof. Clay, I have ordered more by telegraph from the same source.

It appears, therefore, that we are on the eve of a great demand for Chian turpentine. The price is running up at a fabulous rate. and will, no doubt, flood the market with fraudulent imitations.

It is important, therefore, to consider whether any efficient substitute for the real drug can be procured. Chian turpentine is named from "Chios," one of the names of the Island of Scio, where it is produced. It is also found on the Island of Cyprus, and is sometimes called "Cyprus turpentine." Unlike most of the resins, it is not the product of a coniferous tree, but of the *Pistacia terebinthus*, belonging to the natural order *anacardiaceæ*, to which belong also the sumacs, poison ivy (*Rhus tox.*), and *Rhus aromatica*.

In default of the genuine Chian turpentine, it will be rational at present to use the resins of other trees of the same genus, which probably contain similar medicinal properties.

Ordinary mastich is a resin in its physical and chemical qualities closely similar to Chian turpentine, and it is produced from a tree of the same genus, *P. lentiscus*. This drug can be obtained in purity, although somewhat costly, and may be prepared in the same combinations as the Chian turpentine. The latter drug has been used by Professor Clay in the form of an emulsion, giving eight grains of the turpentine and six grains of flowers of sulphur to each dose, three times a day. Mr. E. H. Sargent, 125 State street, of this city, has prepared a confection of mastich and sulphur which seems more likely to be agreeable to many patients than the emulsion.

Whether this new remedy will prove of value, or will go the way of its predecessors into obscurity, is yet to be determined.

Prof. Clay's statements are clear and positive. Some experiments made by myself and by others in this city would seem thus far to corroborate his assertions, but we have not carried them far enough as yet to be able to state whether the apparent improvement will be permanent. An important question arises as to whether Prof. Clay is not mistaken in giving Chian turpentine, rather than sulphur, the primary place in his prescriptions. The malignant element in cancer appears to be the non-adherent, multiplying cells which fill its cavities, and so far as we can judge, its disastrous results are dependent on the enormous power of reproduction of these bodies. If there be a substance in the Materia Medica which can act upon these cells as antiseptics act upon bacteria, by destroying their life, or can modify their vitality, so as to check their multiplication, as arsenic checks the production of epithelial cells in scaly diseases of the skin, such a remedy will cure cancer. Cells already in existence would either be absorbed or remain harmless, if they no longer multiplied.

These cells, in short, suggest to one many analogies with such low forms of organic life as are destroyed by sulphur, arsenic, carbolic acid, etc. The analogy is vague, and not implicitly to be trusted; nevertheless it strongly suggests that the efficacy of this combination may be due to the cell-destroying power of the sulphur or of sulphurous acid, and that the turpentine may be only an adjuvant. Be this as it may, whenever we are unable to obtain genuine Chian turpentine, it would seem best, under the circumstances, to give sulphur in full doses, and to substitute the resin of the *Pistacia lentiscus* for that of the *Pistacia terebinthus*.

The *British Medical Journal* says that the Chian turpentine has been tried in the London Cancer Hospital without any success. It does not state whether the sulphur was used with it or omitted, nor whether an indisputable article of the turpentine was obtained.

NO. 6 SIXTEENTH ST., CHICAGO.

